# Cutting through the weeds: Evaluation of a novel adsorption with crossmatch cells and elution protocol to sharpen HLA antibody identification by the single antigen bead assay

**DOI:** 10.3389/fgene.2022.1059650

**Published:** 2022-11-30

**Authors:** Robert S. Liwski, Sandra Tafulo, Robert Carroll, James H. Lan, Anna L. Greenshields

**Affiliations:** ^1^ Department of Pathology and Laboratory Medicine, Dalhousie University, Halifax, NS, Canada; ^2^ Blood and Transplantation Center of Porto, Portuguese Institute for Blood and Transplantation, Porto, Portugal; ^3^ Health and Medical Sciences, University of South Australia, Adelaide, SA, Australia; ^4^ Transplantation and Immunogenetics Service, Australian Red Cross Blood Services, Adelaide, SA, Australia; ^5^ Department of Pathology and Laboratory Medicine, University of British Columbia, Vancouver, BC, Canada

**Keywords:** HLA antibodies, single antigen bead assay, adsorption, elution, epitopes, denatured antigens, flow cytometry crossmatch, transplantation

## Abstract

The single antigen bead (SAB) assay is the most used test for the identification of HLA specific antibodies pre- and post-transplant. Nevertheless, detection of spurious reactivities remains a recognized assay limitation. In addition, the presence of weak reactivity patterns can complicate unacceptable antigen assignment. This work presents the evaluation of the adsorption with crossmatch cells and elution (AXE) technique, which was designed to help differentiate weak HLA specific antibodies targeting native antigens from spurious and background SAB assay reactivity. The AXE protocol uses selected donor cells to adsorb HLA specific antibodies from sera of interest. Bound antibodies are then eluted off washed cells and identified using the SAB assay. Only antibodies targeting native HLA are adsorbed. Assay evaluation was performed using five cell donors and pooled positive control serum. AXE efficiency was determined by comparing SAB reactivity of adsorbed/eluted antibody to that of the antibodies in unadsorbed sera. A robust efficiency was seen across a wide range of original MFI for donor specific antibodies (DSA). A higher absorption/elution recovery was observed for HLA class I antigens vs. class II. Locus-specific variation was also observed, with high-expression HLA loci (HLA-A/B/DR) providing the best recovery. Importantly, negligible reactivity was detected in the last wash control, confirming that AXE eluates were not contaminated with HLA antibody carry-over. Donor cells incubated with autologous and DSA-containing allogeneic sera showed that AXE selectively adsorbed HLA antibodies in a donor antigen-specific manner. Importantly, antibodies targeting denatured epitopes or other non-HLA antigens were not detected by AXE. AXE was particularly effective at distinguishing weak HLA antibodies from background reactivity. When combined with epitope analysis, AXE enhanced precise identification of antibody-targeted eplets and even facilitated the characterization of a potential novel eplet. Comparison of AXE to flow cytometric crossmatching further revealed that AXE was a more sensitive technique in the detection of weak DSA. Spurious reactivities on the current SAB assay have a deleterious impact on the assignment of clinically relevant HLA specificities. The AXE protocol is a novel test that enables users to interrogate reactive patterns of interest and discriminate HLA specific antibodies from spurious reactivity.

## Introduction

The detection of donor specific antibodies (DSA) targeting Human Leukocyte Antigens (HLA) has been at the fore front of pre- and post-transplant testing ever since the landmark study by Patel and Terasaki was published describing the complement-dependent cytotoxicity crossmatch (Patel and Terasaki, 1969). The sensitivity and specificity of the cytotoxicity crossmatch assay has been improved by the addition of washing steps (Amos et al., 1969), extended incubations (Cross et al., 1977), and enhancement with anti-human globulin (Fuller et al., 1978). The advent of flow cytometry crossmatch (FCXM) (Garovoy et al., 1983; Bray et al., 1989) represented a further improvement in detection of DSA and assessment of pre-transplant immunological risk (Liwski and Gebel, 2018).

A major paradigm change in how HLA specific antibodies are identified occurred when solid phase assays were introduced (Gebel and Bray, 2014). In particular, the single antigen bead (SAB) Luminex assay, which uses purified recombinant HLA antigens conjugated to fluorescently labeled microparticles, detects HLA specific antibodies with exquisite sensitivity and precision and is the most used test for pre- and post-transplant HLA antibody identification and monitoring (Lefaucheur et al., 2008; Amico et al., 2009; Tait et al., 2013). Importantly, the use of the SAB assay allowed the development of the calculated panel reactive antibody (cPRA) metric used for organ allocation (Cecka et al., 2011) and the routine application of virtual crossmatching, enabling national organ sharing and development of kidney paired exchanges (Morris et al., 2019). In addition, it facilitated the identification and characterization of HLA epitopes including TerEps and eplets, and led to the development of epitope-based antibody analysis algorithms used in software such as HLA Matchmaker (Duquesnoy, 2002).

Although the introduction of SAB testing has revolutionized HLA antibody detection and analysis, the assay has several limitations. Limited HLA alleles represented on the SAB panels, variability in antigen density, complement mediated interference with antibody detection, and presence of denatured antigens on the beads can result in false negative and positive reactions making the interpretation challenging (Middelton et al., 2014; Visentin et al., 2015). Importantly, there is now widespread recognition and concern that SAB assays frequently detect spurious antibody reactivities that are not clinically relevant. The cause of these observations is likely multifactorial. One intrinsic assay factor may relate to the conjugation of denatured HLA antigens to microparticle beads during manufacturing, resulting in the unintended detection of antibodies that bind to cryptic targets of denatured proteins rather than to HLA epitopes in their natural conformation (Morales-Buenrostro et al., 2008; Cai et al., 2009; El-Awar et al., 2009; Oaks et al., 2014). Visentin et al. (Visentin et al., 2014) reported 39% of wait listed patients showed evidence of antibodies to denatured antigens. These antibodies were deemed clinically insignificant based on negative FCXM results but were listed as unacceptable antigens, thus hindering candidates’ access to transplantation.

In 2007, El-Awar described an elegant method for isolating HLA antibodies using adsorption-elution with recombinant single HLA antigen cell lines and testing the eluate with the SAB assay (El-Awar et al., 2007; El-Awar et al., 2017). This method helped define HLA antibody specificity and characterize epitopes on HLA. Building on this work with the goal to overcome the major challenges associated with current HLA antibody detection and analysis, we developed and optimized a novel protocol named adsorption with crossmatch cells and elution (AXE) technique, which specifically detects antibodies that show evidence of binding to native HLA molecules on the cell surface. In this study, we evaluate the AXE procedure for adsorbing eluting HLA specific antibodies from sera and highlight its clinical utility in improving the identification and analysis of HLA antibodies.

## Materials and methods

### Reagents

All washes in the AXE procedure, including those conducted during donor cell preparation and following the antibody adsorption procedure, were performed using phosphate buffered saline (PBS; Life Technologies Inc., Burlington ON, Canada). Antibody eluates were prepared using the acid eluting solution (solution I; ELU KIT^TM^ Plus; Immucor, Dominion Biologicals Limited; Dartmouth, Canada) and the eluate pH was neutralized (6.8–7.2 range) using the base buffering solution (Solution II; ELU KIT^TM^ Plus; Immucor, Dominion Biologicals Limited). The pH was verified using plastic pH indicator strips (Thermo Fisher Scientific Inc.). SAB assays were performed using LABScreen SAB kits (LS1A04 lot 13 for HLA class I and LS2A01 lots 14 and 15 for HLA class II; One Lambda, Canoga Park, CA), phycoerythrin (PE)-conjugated goat anti-human IgG (LS-AB2; One Lambda), LABScreen wash buffer (LWB; One Lambda) and 96-well V-bottom trays (Whatman Pistcataway, NJ). FCXM were set up in 96-well U-bottom BD Falcon Microplate trays (BD Biosciences) and washes were performed with flow wash buffer (FWB) composed of phosphate buffered saline (PBS; Life Technologies Inc.) and 2% (v/v) fetal calf serum (Life Technologies Inc.). Anti-CD3-PerCP (clone SK7) and anti-CD19-PE (clone SJ25C1) monoclonal antibodies were purchased from BD Biosciences (Mississauga, ON, Canada). Fluorescein (FITC) conjugated F (ab’)_2_ fragment goat anti-human IgG, Fcγ specific polyclonal antibody (IgG-FITC), 1.0 mg/ml stock solution, was purchased from Jackson ImmunoResearch Laboratories Inc. (West Grove, PA). Lymhocytes were isolated with the EasySep^TM^ Direct Human Total Lymphocyte Isolation Kit (EasySep^TM^ Direct; STEMCELL Technologies Inc., Vancouver, BC, Canada) and were treated with pronase (4.7 units/mL; Sigma-Aldrich, St Louis, MO) and DNase (11,000 units/mL; Sigma-Aldrich).

### Sera and donor cell selection

Cells for all AXE evaluation studies were obtained from acid dextrose citrate (ACD) anticoagulated whole blood samples collected from volunteers as well as live and deceased donors in accordance with the institutional assay validation protocol. Pooled positive control sera (PPC; a pool of 20 highly sensitized patient sera, cPRA >95%) were diluted to 1:128 and 1:8 in PBS for AXE protocol evaluation for class I and class II HLA antibodies, respectively. LABScreen negative control sera (One Lambda) were used in all SAB assay testing. Sera from patients awaiting solid organ transplantation were used to evaluate the AXE procedure. The ability of AXE to detect antibodies targeting native HLA epitopes was evaluated in sera containing genuine HLA reactivities that have been confirmed on cell-based crossmatches (n=6). In addition, sera with known spurious reactivity patterns (*n* = 3) on the SAB assay were tested to assess the assay’s specificity. All sera were treated with EDTA disodium salt solution at a final concentration of 6.0 mM (0.5 M stock solution, Cat# E7889; Sigma-Aldrich, St Louis, MO), before AXE or SAB testing.

### Cell isolation for the AXE procedure

ACD anticoagulated whole blood samples (6 ml) were centrifuged at 1800 x g for 10 min with no brake. Buffy coat layers (200 μl) were collected, placed into a 1.5 ml microfuge tube and resuspended in 1.2 ml of PBS. Cells were washed twice in PBS by centrifugation at 800 x g for 1 min and then resuspended in 1 ml of PBS.

### AXE protocol

The isolated donor cell suspension (1 buffy coat equivalent) was divided equally into two 1.5 ml microfuge tubes, centrifuged at 800 x g for 1 min and the supernatants were removed. Two hundred μl of EDTA treated test serum was added to the donor cell pellet in the first microfuge tube while PPC was added to the second tube. Serum/cell suspensions were mixed well by pipetting up and down. Tubes were placed into a 37°C heat block and incubated for 30 min. One ml of PBS was added to each tube and the cells were washed 6 times by centrifugation at 800 x g for 1 min. On the last wash 100 μl of the supernatant was collected from each tube and placed into a clean tube. This supernatant served as the last wash control to ensure no significant amount of unbound HLA antibodies were present prior to the elution procedure. Next, 50 μl of the acid eluting solution (solution I; ELU KIT^TM^ Plus) were added to the dry cell pellets and the tubes were gently vortexed. After a 1 min incubation at room temperature (RT), tubes were centrifuged at 800 x g for 1 min, then 40 μl of eluate was carefully removed and placed in a clean tube containing 50 μl of the base buffering solution (Solution II; ELU KIT^TM^ Plus) to neutralize the pH (6.8–7.2). The appropriate pH range was verified by placing 1 μl of each on the Fisherbrand Plastic pH strip. All eluates and last wash controls were immediately tested for HLA antibodies using the LABScreen SAB assay.

### SAB assay testing using the rapid optimized SAB protocol

All SAB assays were performed using the ROB protocol as previously described (Liwski et al., 2017). Briefly, 20 μl of EDTA (6.0 mM) treated sera, eluate or last wash control samples was added to appropriate wells of 96-well V-bottom trays containing either class I or class II HLA LABSCreen single antigen beads. Trays were incubated for 15 min at RT and washed 4 times in Luminex wash buffer by centrifugation at 1800 x g for 1 min. After the last wash, 20 μl of anti-IgG-PE (1:10 dilution in PBS) secondary antibody was added to each well and the trays were incubated for 5 min at RT in the dark. Following two additional washes, beads were resuspended in 55 μl of LABScreen wash buffer and samples were acquired using Luminex 3D instruments. The results were analysed with Fusion software version 4.3.1 using the baseline MFI formula.

### The Halifaster FCXM protocol

FCXMs were performed using the Halifaster protocol as previously described (Liwski et al., 2018). Briefly, 30 µl of test or control sera and 15 µl of purified (EasySep Direct) donor lymphocytes (1.5 × 10^5^ cells) were added to each reaction well in a 96-well U-bottom BD Falcon Microplate tray, dry vortexed, and incubated at RT for 20 min. Cells were washed three times with 200 µl FWB at 500 x g for 1 min, after which antibody cocktail (2 µl of anti-CD3-PerCP, 1 µl of anti-CD19-PE, 0.125 µl anti-IgG-FITC, made up to 50 µl with PBS) was added to the cells, dry vortexed, and incubated for 5 min at RT in the dark. Cells were washed twice more with 200 µl FWB at 500 x g for 1 min, resuspended in 150 μl FWB, and transferred into 5 ml polystyrene Falcon tubes containing an additional 250 μl of FWB before acquisition on the BD FACSCanto II flow cytometer.

### Statistical analysis

AXE protocol efficiency at recovering HLA antibodies was calculated as a percentage of the SAB assay mean fluorescence intensity (MFI) seen for the HLA specificities in the eluate as compared to the original serum sample. Mean and standard deviation calculations were performed using Microsoft Excel software.

## Results

### Evaluation of the adsorption with crossmatch cells and elution procedure

**FIGURE 1:**
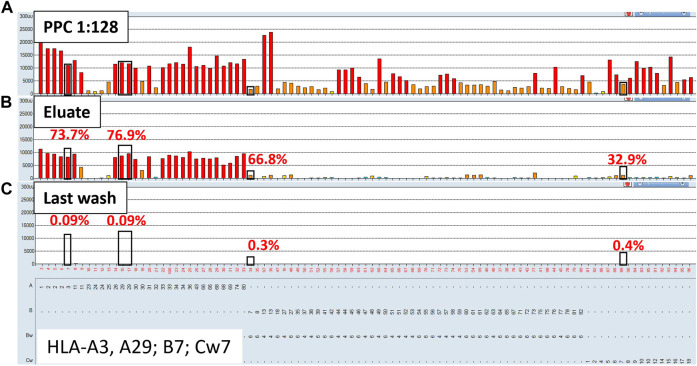
Class I HLA antibodies are efficiently adsorbed/eluted from sera using the AXE protocol. Representative SAB images of Class I HLA specificities in the pooled positive control (PPC; 1:128 dilution) serum **(A)**, the AXE eluate **(B)** and the last wash control **(C)** are shown. Mean fluorescence intensity (MFI) values are displayed on the Y axis. The SAB HLA specificities, *X* axis of panel **(C)** are sorted in numerical order within each HLA locus (HLA-A, B and C). Bead reactions corresponding to the AXE donor specific antigens are highlighted with black rectangles and the antigens are listed at the bottom of panel **(C)**. The top of each black rectangle aligns with the maximum PPC MFI value in the 1:128 diluted PPC serum **(A)**. The efficiency (% MFI compared to serum) of adsorption/elution by AXE **(B)** and the residual reactivity (% MFI compared to serum) in the last wash control **(C)** are indicated with red font.

**FIGURE 2:**
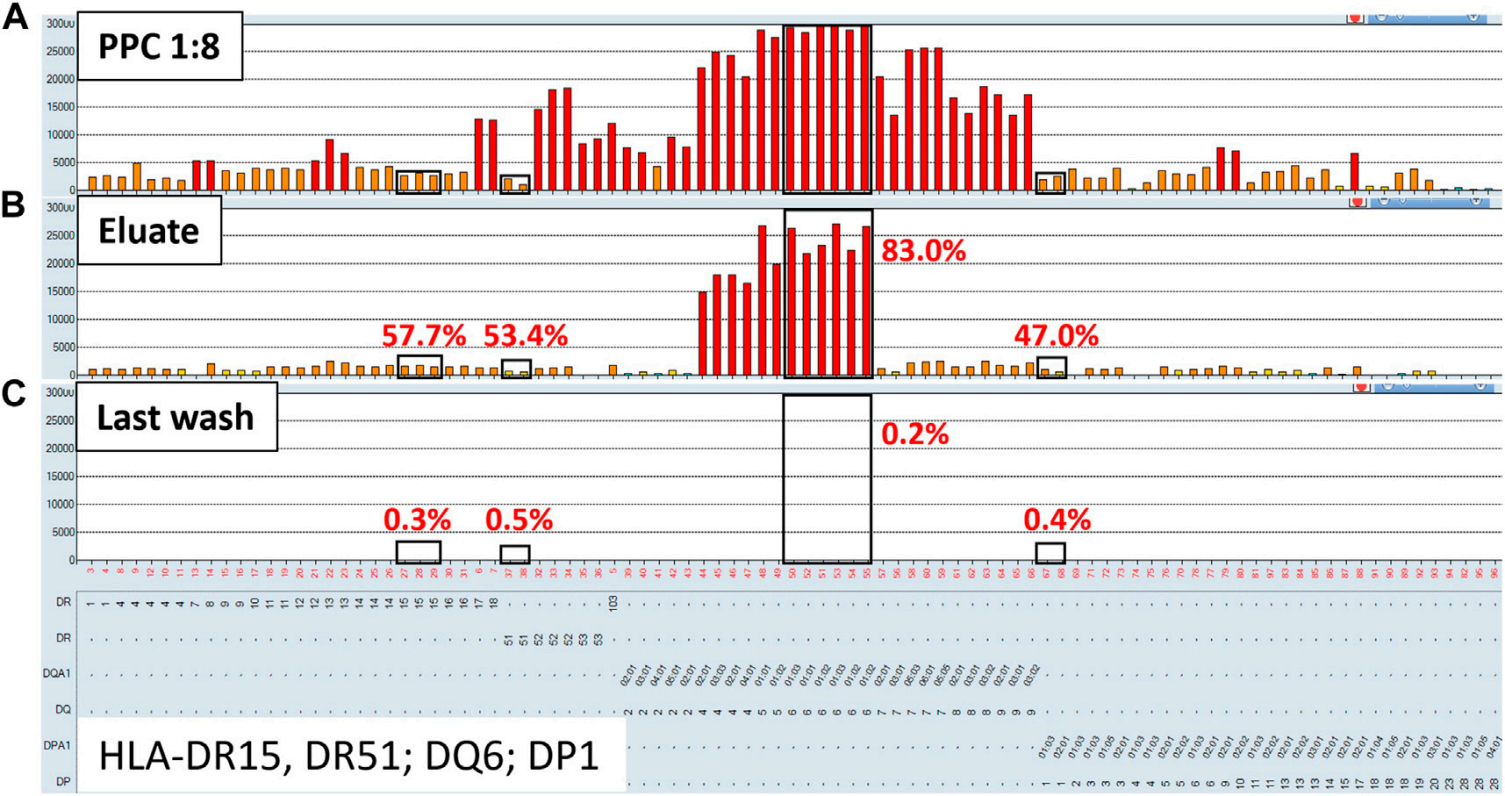
Class II HLA antibodies are efficiently adsorbed/eluted from sera using the AXE protocol. Representative SAB images of Class II HLA specificities in the pooled positive control (PPC; 1:8 dilution) serum **(A)**, the AXE eluate **(B)** and the last wash control **(C)** are shown. Mean fluorescence intensity (MFI) values are displayed on the Y axis. The SAB HLA specificities (*X* axis of panel **(C)** are sorted in numerical order within each HLA locus (HLA-DR, DQ and DP). Bead reactions corresponding to the AXE donor specific antigens are highlighted with black rectangles and the antigens are listed at the bottom of panel **(C)**. The top of each black rectangle aligns with the maximum PPC MFI value in the 1:8 diluted PPC serum **(A)**. The efficiency (% MFI compared to serum) of adsorption/elution by AXE **(B)** and the residual reactivity (% MFI compared to serum) in the last wash control **(C)** are indicated with red font.

To evaluate the AXE procedure for its efficiency of recovering HLA antibodies from sera, buffy coat cells isolated from healthy donor blood samples (*n* = 5) were used to individually adsorb and elute HLA antibodies from the pooled positive control (PPC) sera. The PPC was generated by pooling sera from 20 highly sensitized (cPRA ≥95%) transplant candidates to ensure strong reactivity with all HLA antigens represented on the LABScreen class I and class II HLA SAB panels. For the AXE procedure evaluation, the PPC serum was diluted to 1:128 for class I HLA (Figure 1A) and 1:8 for class II HLA (Figure 2A) to test the efficiency of adsorption and elution of HLA antibodies over a broad range of mean fluorescence intensity (MFI) values. The last wash control, which is the supernatant collected after the final wash following the adsorption portion of the procedure, was used to ensure that all unbound antibodies were eliminated prior to the elution process.

**FIGURE 3:**
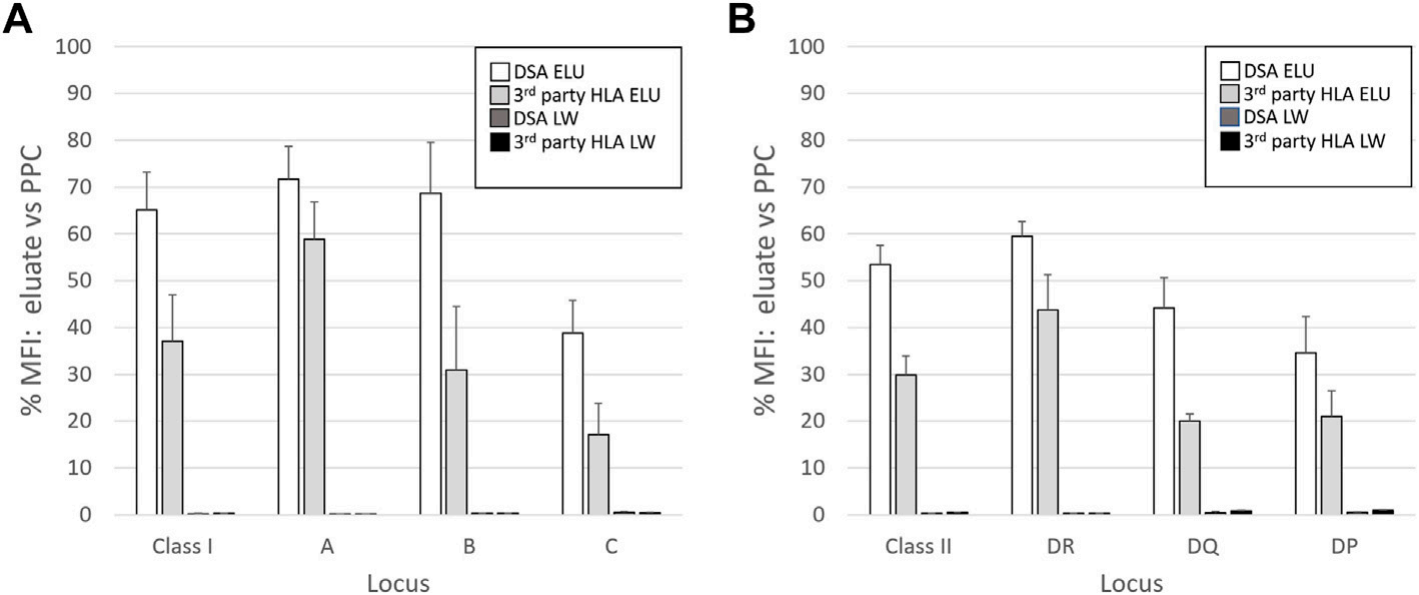
The efficiency of adsorption/elution by AXE is HLA locus dependent. The mean efficiency (% MFI compared to serum) of AXE adsorption/elution of DSA (DSA ELU; white bars) and 3^rd^ party HLA specificities (3rd party HLA ELU; light grey bars), as well as the mean residual reactivity (% MFI compared to serum) in the last wash controls for the DSA (DSA LW; dark grey bars) and 3^rd^ party HLA specificities (3^rd^ party HLA LW; black bars) are shown for class I **(A)** and class II **(B)** locus specific HLA antibodies. The results are a mean +/- SD of separate experiments in which the AXE method was used to adsorb/elute HLA antibodies from PPC sera (1:128 dilution for class I and 1:8 dilution for class II HLA) with five different donor cells.

Representative class I HLA SAB assay histograms of AXE evaluation studies performed using the PPC and one of the donor cells are shown in Figure 1. For ease of visualization, the HLA antigens in each SAB panel were sorted in numerical order within each locus (Figure 1). Black rectangles highlight the AXE donor cell HLA antigens (HLA-A*03:01, *29:02; B*07:02; C*07:02) with the top of each rectangle aligning with the maximum PPC MFI values of antibodies specific to the corresponding AXE donor cell antigens. The efficiency of adsorption/elution of the AXE procedure was expressed as a percentage of donor specific antibody (DSA) MFI recovered in the eluate vs. serum sample and was as follows: HLA-A3 = 73.7%, A29 = 76.9%, B7 = 66.8%, and Cw7 = 32.9% (Figure 1B). Interestingly, the efficiency of AXE when eluting antibodies targeting donor antigens was similar across a wide range of antibody MFI. For example, the AXE efficiency was comparable when eluting antibodies targeting HLA-B7 and HLA-A3/A29 donor antigens despite the significantly lower starting MFI values for HLA-B7 (MFI = 2,027) compared to HLA-A3 (MFI = 11,300) or HLA-A29 (average MFI = 12,220) in the PPC sample (Figures 1A,B). In contrast, the percent MFI recovered by AXE for antibodies binding to 3^rd^ party HLA antigens was variable (range: 1.01%–77.9%). For example, while HLA-A1 specific antibodies eluted at 49.5% of the PPC MFI (PPC MFI = 23,000; eluate MFI = 11,450, Figures 1A,B), HLA-B13 specific antibodies eluted at an average of only 4.7% (mean PPC MFI = 23,600; mean eluate MFI = 1,120; Figures 1A,B). This is likely related to the epitope specificity of eluted antibodies and the differential degree to which donor-specific epitopes are shared on 3^rd^ party HLA antigens. Importantly, the percentage of MFI remaining in the last wash control was negligible for both donor-specific (range: 0.09%–0.4%; eluate MFI range: 6.2–16.3; Figure 1C) and 3^rd^ party (range: 0.04%–1.7%; eluate MFI range: 5.1–25.5; Figure 1C) antigens, demonstrating that the antibodies eluted by AXE were those specifically adsorbed out by the donor cell HLA antigens.

Representative histograms for the class II HLA AXE procedure evaluation are shown in Figure 2. The percentage of DSA MFI recovered in the eluate following AXE procedure was 57.7% for HLA-DR15, 53.4% for DR51, 83.0% for DQ6 and 47.0% for DP1 (Figure 2B). Thus, the overall efficiency of AXE was excellent despite the relatively low starting MFI of DSA in the PPC sample for some of the class II HLA specificities (PPC mean MFI: DR15 = 2,930; DR51 = 1,875 and DP1 = 2,570; Figure 2A). As seen for class I HLA, the efficiency of the AXE protocol in adsorbing/eluting antibodies directed against 3^rd^ party class II HLA antigens was variable (range 0.4%–90.2%; Figure 2B). Importantly, HLA antibody reactivity observed in the last wash control was negligible (Figure 2C).

The overall results of AXE protocol evaluation with all five donor cells are presented in Figure 3. On average, antibodies against class I HLA antigens appeared to be adsorbed/eluted slightly better compared to those binding to class II HLA (65.1% vs. 53.2%; Figures 3A,B). Within class I, antibodies targeting HLA-A and B antigens appeared to be adsorbed/eluted more efficiently compared to those targeting HLA-C loci (71.7% for HLA-A, 68.6% for HLA-B, 38.8% for HLA-C; Figure 3A). Such locus specific differences in the efficiency of AXE protocol were also seen for class II HLA with HLA-DR specific antibodies being adsorbed/eluted most efficiently followed by HLA-DQ and HLA-DP (59.5% for HLA-DR, 44.2% for HLA-DQ, and 34.5% for HLA-DP Figure 3B). These differences in adsorption/elution efficiency are likely related to locus specific variation in HLA expression on leukocytes and the fact that a smaller percentage of leukocytes in blood express class II HLA when compared to class I. Finally, the level of HLA antibodies targeting both donor specific and 3^rd^ party HLA antigens detected in the last wash control samples was negligible.

### The AXE procedure mediates adsorption and elution in a donor antigen specific manner

**FIGURE 4:**
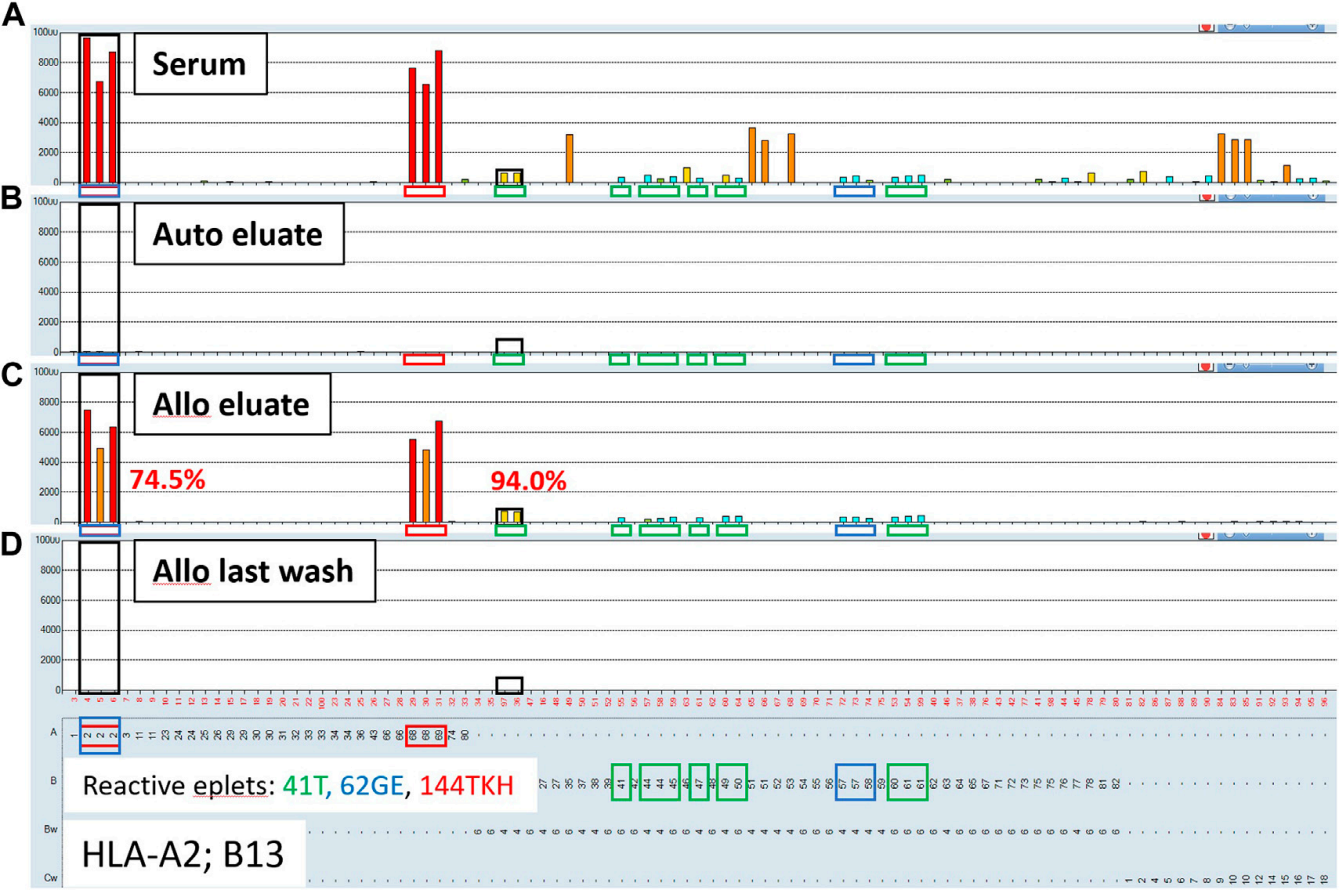
Adsorption/elution of HLA antibodies by AXE is donor antigen specific. Representative SAB images of Class I HLA specificities in a sensitized patient’s serum **(A)**, the AXE eluate using autologous cells **(B)**, the AXE eluate using allogeneic cells **(C)** and the allogeneic cell last wash control **(D)** are shown. Mean fluorescence intensity (MFI) values are displayed on the Y axis. The SAB HLA specificities (*X* axis) are sorted in numerical order within each HLA locus (HLA-A, B and C). Bead reactions corresponding to the AXE donor specific antigens (A*02:01 and B*13:02) are highlighted with black rectangles and the antigens are listed at the bottom of panel **(D)**. The top of each black rectangle aligns with the maximum MFI value in the patient’s serum **(A)**. HLA antigens expressing the mismatched donor 41T, 62GE and 144TKH eplets are highlighted using green, blue and red rectangles, respectively. The efficiency (% MFI compared to serum) of adsorption/elution by AXE using allogeneic cells **(C)** is indicated with red font.

To confirm that the AXE procedure selectively adsorbs/elutes HLA antibodies in an antigen specific manner, sera from sensitized patients (*n* = 3) were adsorbed/eluted using autologous cells (auto eluate) vs. surrogate allogeneic donor cells (allo eluate) expressing HLA antigens targeted by antibodies in sensitized patients’ sera. The eluates and the corresponding last wash controls were then tested by the SAB assay. Representative results from these experiments are shown in Figure 4 and demonstrate that while HLA antibodies cannot be adsorbed/eluted from sera using autologous cells (auto eluate; Figure 4B), allogeneic donor cells expressing HLA-A2 and B13 antigens adsorbed HLA antibodies from the same serum in a donor antigen specific fashion (allo eluate; Figure 4C). In fact, the HLA antibody reactivity pattern seen in the allogeneic donor cell eluate is explained with three eplets expressed by donor antigens: 41T (B13, 41, 44, 45, 47, 49, 50, 60 and 61; green squares; Figure 4), 62 GE (A2, B57, B58; blue squares; Figure 4) and 144TKH (A2, 68, 69; red squares; Figure 4). The adsorption/elution efficiency in this case was high (74.5% for HLA-A2 and 94% for HLA-B13 DSA; Figure 4C), despite the low starting MFI for the B13 antibody in serum (mean MFI = 640; Figure 4A). Interestingly, several relatively strong 3^rd^ party HLA-B and C locus antibody specificities (such as B35, B53, Cw9, Cw10 and Cw15) seen in the original serum sample were not adsorbed/eluted with the allogeneic donor cells. The eplet analysis shows that these specificities share the 94I eplet, indicating that these were genuine and not spurious reactivities. Because the target 94I eplet is not expressed on either A*02:01 or B*13:02 donor alleles, the results further illustrate the specificity of the AXE protocol as indicated by the absence of the 3^rd^ party specificities when adsorbed/eluted using the allogeneic donor cells. Finally, the SAB assay performed on the allogeneic donor last wash control was completely negative, demonstrating that no unbound HLA antibodies remained in the supernatant prior to the elution procedure (Figure 4D).

### Antibodies targeting denatured epitopes or non-HLA contaminants present on the single antigen beads are not adsorbed and eluted using the AXE procedure

**FIGURE 5:**
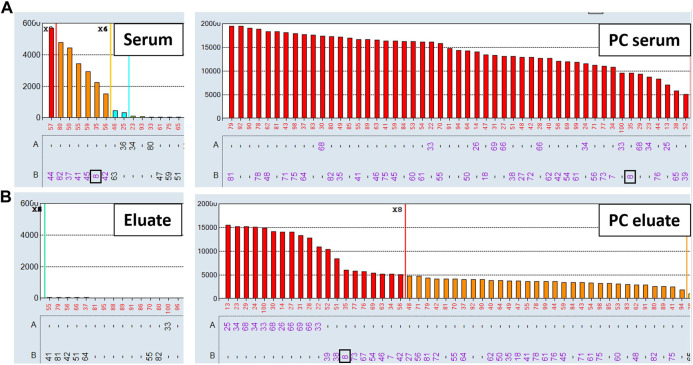
Antibodies targeting the 156D denatured epitope are not adsorbed/eluted with the AXE protocol. SAB images of Class I HLA specificities in a patient’s serum **(A)**, (left panel) and the positive control serum **(A)**, (right panel), as well as AXE eluates from patient’s serum **(B)**, (left panel) and from the positive control serum **(B)**, (right panel) using HLA-B8 mismatched donor cells are shown.

**FIGURE 6:**
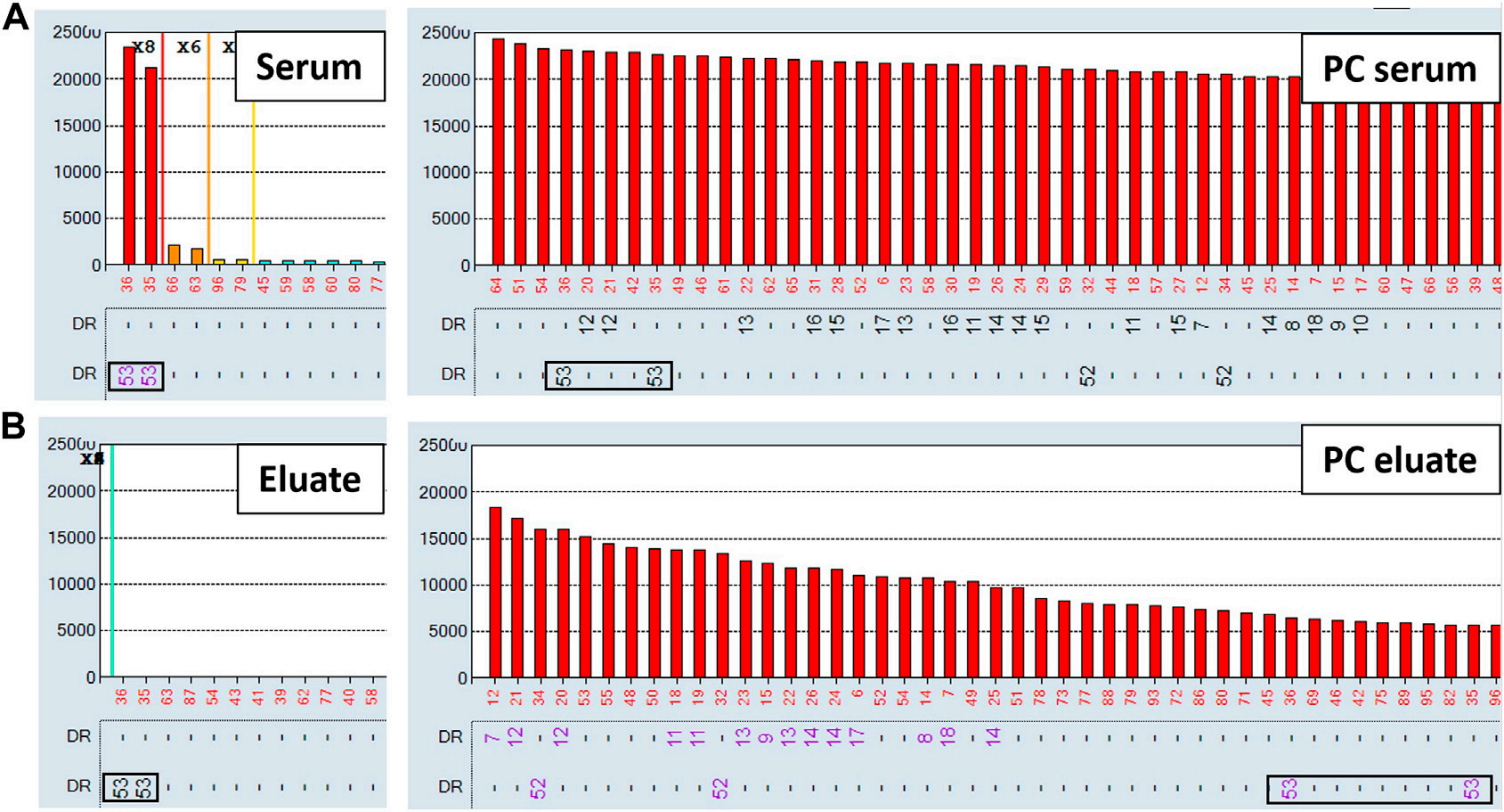
Spurious antibodies targeting HLA-DR53 expressing SABs are not adsorbed/eluted with the AXE protocol. SAB images of Class II HLA specificities in a patient’s serum **(A)**, (left panel) and the positive control serum **(A)**, (right panel), as well as AXE eluates from patient’s serum **(B)**, (left panel) and from the positive control serum **(B)**, (right panel) using HLA-DR53 mismatched donor cells are shown.

**FIGURE 7:**
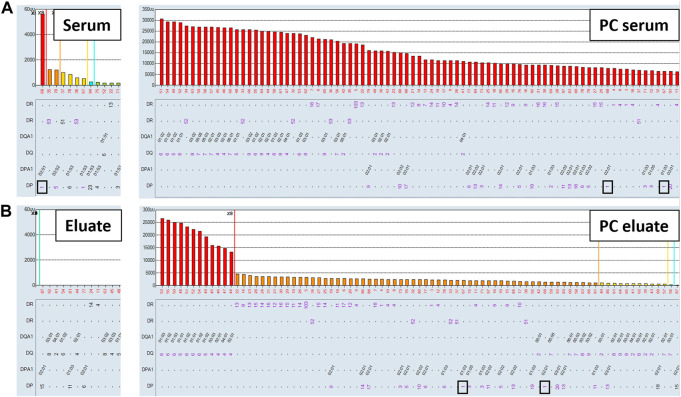
Spurious antibody reactivity targeting HLA-DP1 expressing SABs is not adsorbed/eluted with the AXE protocol. SAB images of Class II HLA specificities in a patient’s serum **(A)**, (left panel) and the positive control serum **(A)**, (right panel), as well as AXE eluates from patient’s serum **(B)**, (left panel) and from the positive control serum **(B)**, (right panel) using HLA-DP1 mismatched donor cells are shown.

Non-specific antibodies directed against denatured HLA epitopes or other non-HLA targets on the single antigen beads can give positive reactions in the SAB assay but are not predicted to be adsorbed and eluted using donor cells expressing native HLA antigens. To this end, we performed the AXE protocol on three sera with known spurious reactivity patterns on the SAB test. Figure 5A depicts the class I HLA SAB assay histogram showing a moderately strong antibody reactivity pattern consistent with the 156D epitope (HLA-B8, B37, B41, B42, B*44:02, B45 and B82). This epitope was previously identified as a denatured epitope (El-Awar et al., 2009), which was also confirmed by others using acid denaturation of class I SAB (Eapen et al., 2011). As expected, AXE adsorption/elution using HLA-B*08:01 positive donor cells demonstrated no evidence of antibody binding to native HLA on the cell surface (eluate; Figure 5B). In contrast, HLA-B8 specific antibodies were adsorbed/eluted from PPC using the same donor cells with the efficiency of approximately 58% (PC serum MFI = 9,500, PC eluate MFI = 5,500; Figure 5). Another relatively common artefact seen with the LABScreen class II SAB panel is a strong reactivity with the HLA-DR53 expressing beads (Figure 6A). The AXE procedure demonstrated that these antibodies were not adsorbed/eluted using HLA-DR53 expressing donor cells (Figure 6B; eluate). The efficiency of AXE for adsorbing/eluting DR53 reactivity from PPC was 30% in this experiment (Figure 6; PC serum vs. PC eluate). Finally, Figure 7A shows a frequently observed artefact on the LABScreen class II HLA SAB assay involving DP1, DP5 and DR53 expressing beads which was shown to be non-reactive against HLA-DP1 expressing donor cells (Figure 7B; eluate), confirming that these antibodies do not bind to native HLA-DP1 antigen. Importantly, DP1 specific antibodies were adsorbed/eluted from PPC using the same donor cells with the efficiency of approximately 25% (Figure 7; PPC serum and PPC eluate).

### The utility of the AXE procedure in distinguishing low level HLA antibodies from background reactivity seen with the single antigen bead assay

**FIGURE 8:**
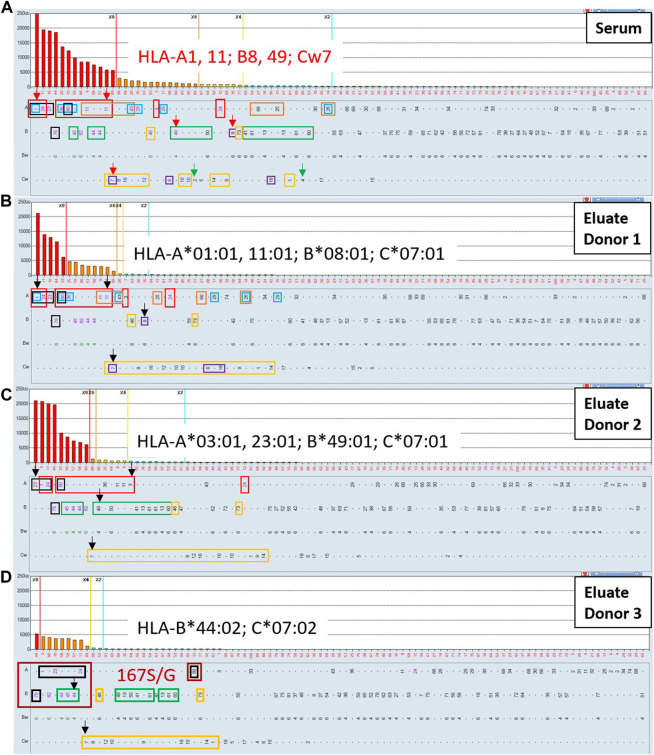
The AXE protocol allows for accurate characterization of post-transplant class I HLA antibody patterns including weak antibody reactivities. SAB images of Class I HLA specificities in a post-transplant sensitized patient’s serum **(A)** and the AXE eluate using cells from surrogate donor 1 **(B)**, donor 2 **(C)** and donor 3 **(D)** are shown. Mean fluorescence intensity (MFI) values are displayed on the Y axis. The SAB HLA specificities (*X* axis) are sorted in order of decreasing MFI. Previous transplant donor mismatched antigens are listed in red font and indicated with red arrows **(A)**. Patient’s self HLA antigens are indicated with green arrows **(A)**. Mismatched donor specific alleles for the AXE donor 1, donor 2 and donor 3 are listed in each panel in black font and indicated with black arrows **(B–D)**. HLA antigens expressing the relevant mismatched donor eplets: (76ANT blue, 144TKR red, 163RW orange, 166DG black, 9D purple, 41T green, and 76VRN yellow rectangles) are indicated. The specificities expressing novel 167S/G epitope are highlighted with dark red rectangles **(D)**.

In this section we highlight the utility of the AXE procedure in confirming the presence of low-level HLA antibodies in clinical samples where such antibodies could be difficult to distinguish from non-specific background reactivities. Figure 8A shows the class I HLA antibody SAB histogram from a highly sensitized renal transplant candidate relisted a few years after failing his first deceased donor transplant. Single antigen beads corresponding to patient’s own HLA typing and the previous transplant donor HLA typing are indicated with green and red arrows, respectively. In this case there is no clear separation of positive signals from background on the histogram. If a commonly accepted MFI threshold of 2,000 MFI is used to define unacceptable antigens (HLA-A1, A11, A23, A*24:02, A*29:01, A36, A43, A80, B44, B45, B76, B82, Cw7, Cw8 and Cw16), this patient would have an elevated cPRA of 88%. If a more stringent 1,000 MFI threshold is applied, additional positive specificities include HLA-A3, A*29:02, B46, Cw6, Cw10 and Cw12, further increasing the cPRA to 92%. When eplet analysis, a more biologically rational approach to antibody assessment, is performed using the previous donor antigens (A1, B8, B49, Cw7) to identify mismatched eplets from the first transplant (76ANT blue, 144TKR red, 163RW orange, 166DG black, 9D purple, 41T green, and 76VRN yellow rectangles), it is clear that many weak antibody specificities reacting below the 1,000 MFI cutoff could have arisen as a result of transplant related sensitization, thereby posing an immune risk to repeat transplantation. AXE studies of this serum using two distinct donor cells expressing among them all the relevant mismatched antigens and eplets (donor 1: A*01:01 A*11:01, B*08:01, C*07:01; Figure 8B and donor 2: A*03:01, A*23:01, B*49:01, C*07:01; Figure 8C) were able to adsorb/elute the entire reactivity pattern, including all specificities below 1,000 MFI in the original serum, in an antigen/eplet specific manner (Figures 8B,C). Importantly, neither the Cw2 nor Cw4 weak “autologous” reactivities seen with the SAB testing of the original serum (Figure 8A) were eluted from either donor cell (Figures 8B,C), confirming the specificity of the adsorption/elution testing.

Interestingly, the eluate from donor 1 (Figure 8B) exhibited a strong reactivity with B44, B45 and B82 antigens, which was unexpected based on the eplet analysis. The B44 and B45 specificities were explained in the original serum by the 41T eplet shared with the immunizing B49 antigen, however, this eplet was not present on any of the AXE donor one mismatched antigens. In addition, the B82 reactivity could not be explained in the original serum using eplet analysis in the context of transplant related sensitization. These findings suggest that the antibodies specific to B44, B45 and B82 antigens eluted in this AXE study are targeting a novel epitope. Indeed, the amino acid sequence alignment revealed the presence of a glycine (G) or serine (S) at position 167 that could explain these results. This polymorphic position is shared by two existing eplets: 166DG (A1, A23, A*24:02, A80 and B76) and 163LS/G (B44, B45, B76 and B82) and the adsorbing antigen from which the entire reactivity was eluted in this case was A*01:01. To confirm this novel epitope, an HLA-B*44:02 mismatched donor which carries this novel epitope was used for additional AXE studies (Figure 8D). The results confirmed that HLA-B*44:02 alone could adsorb the entire pattern of reactivity including A1, A23, A*24:02, A80, B45, B76 and B82. As expected, all specificities expressing the 41T (B*44:02 mismatch) eplet and 76VRN (C*07:02 mismatch) eplet were also adsorbed and eluted with this donor cell. Importantly, last wash AXE controls were negative with all surrogate donor cells (not shown).

**FIGURE 9:**
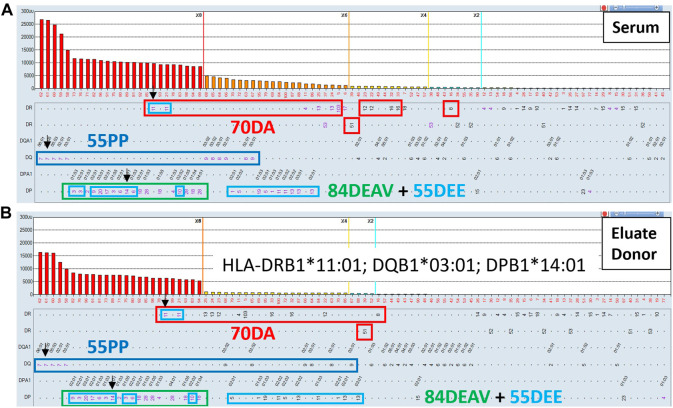
The AXE protocol allows for accurate characterization of class II HLA antibody patterns including weak antibody reactivities. SAB images of Class II HLA specificities in a sensitized patient’s serum **(A)** and the AXE eluate using cells from a surrogate donor **(B)**, are shown. Mean fluorescence intensity (MFI) values are displayed on the Y axis. The SAB HLA specificities (*X* axis) are sorted in order of decreasing MFI. Mismatched AXE donor specific antigens are listed in panel B and are indicated with black arrows **(A and B)**. HLA antigens expressing the relevant mismatched donor eplets: (70DA red, 55 PP dark blue, 55DEE light blue and 84DEAV green rectangles) are indicated.

Figure 9 illustrates an example where the AXE protocol could complement eplet analysis to define clinically relevant, low-level class II antibodies in a highly sensitize patient with a history of multiple pregnancies. The SABs corresponding to patient’s HLA typing are indicated with green arrows. The eplet analysis suggested that four distinct eplets, namely: 70DA (red rectangles), 55 PP (dark blue rectangles) 84DEAV (green rectangles) and 55DEE (light blue rectangles) can explain most of the reactivity in this serum. Importantly, several of the HLA-DR specificities (DR103, DRB1*04:02, DR8, DR12, DR13, DR14, DR16 and DRB5*01) accounted for by the eplet analysis are below the commonly used 2,000 and 1,000 MFI thresholds and could be interpreted as background reactivity by many HLA laboratories. However, the AXE protocol studies using cells from a donor mismatched for 3 antigens of interest (DRB1*11:01, DQB1*03:01, DQA1*05:05, DPB1*14:01) expressing the four relevant eplets clearly adsorbed and eluted the entire expected reactivity pattern including the low-level DR specific antibodies (Figure 9B). Importantly, the SAB assay performed on the last wash control (not shown) was completely negative confirming that antibodies in this serum were targeting native HLA epitopes.

### Comparison of AXE Assay’s sensitivity for antibody detection with flow cytometric crossmatches

**FIGURE 10:**
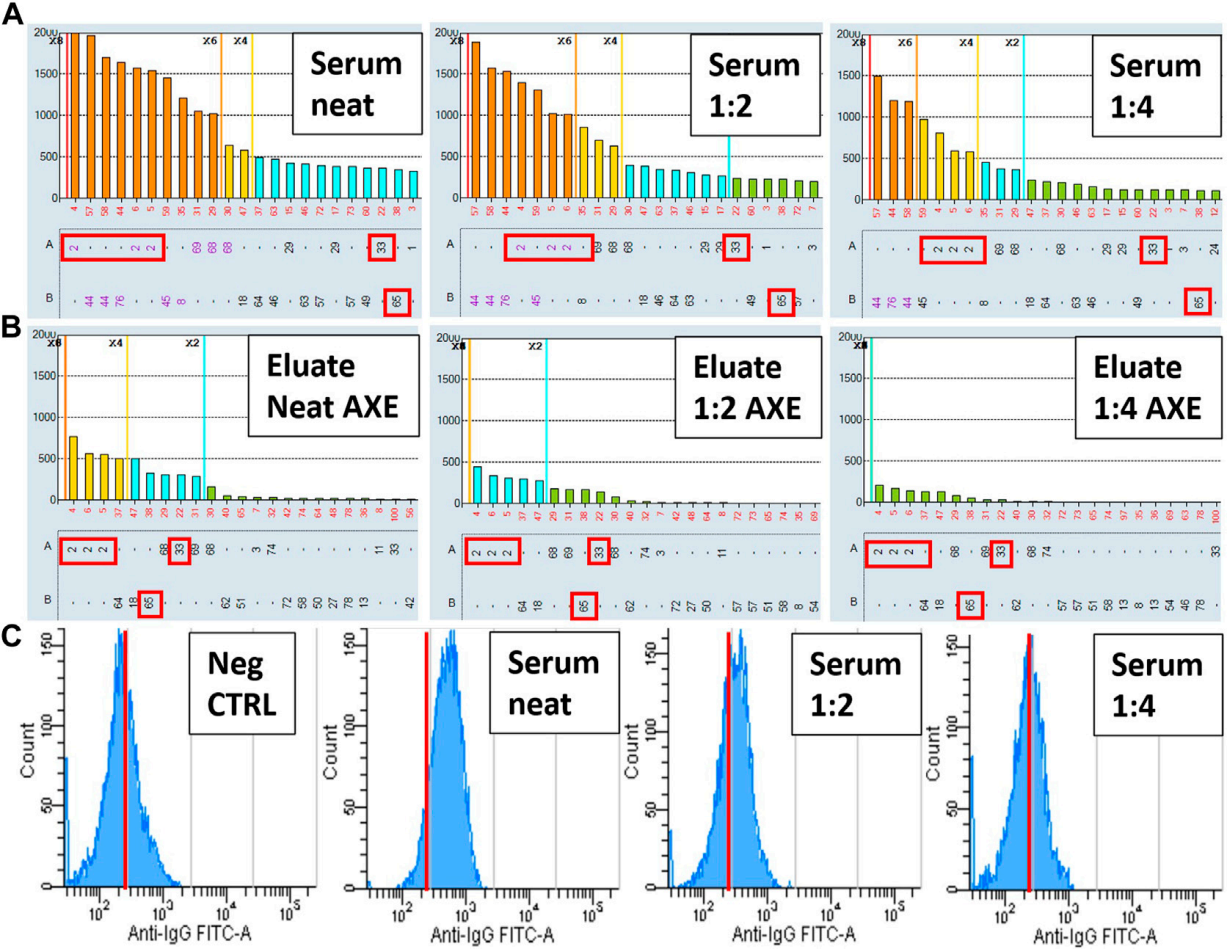
The AXE protocol exhibits increased sensitivity and provides improved specificity compared to the FCXM assay. SAB images of Class I HLA specificities in a sensitized patient’s serum **(A)** and the AXE eluates using cells from a surrogate donor **(B)**, are shown. Serum was tested by SAB and adsorbed/eluted using the AXE protocol at three concentrations (neat, 1:2 and 1:4). Mean fluorescence intensity (MFI) values are displayed on the Y axis **(A and B)**. The SAB HLA specificities (*X* axis) are sorted in order of decreasing MFI **(A and B)**. Mismatched AXE donor specific antigens (HLA-A2, A33, B65) are indicated with red rectangles **(A and B)**. Panel C depicts histograms of T cell FCXM performed against the AXE donor cells using the negative control serum (left panel) and three concentrations of patient’s serum (neat, 1:2 and 1:4). Number of cellular events is displayed on the Y axis and the anti-IgG FITC median fluorescence intensity is shown on the *X* axis. In each histogram **(C)** red line cutoffs are set at 250 median fluorescence intensity units to facilitate visualization of fluorescence shifts relative to the negative control serum.

In Figure 10 we show that the AXE protocol has superior sensitivity in detecting low level HLA antibodies compared to surrogate crossmatches. In this example involving a pregnancy-sensitized patient with a known weak class I HLA antibody reactivity pattern to mismatched HLA-A2 and B44 spousal antigens, flow cytometric T cell crossmatches against a surrogate donor (with HLA-A2, A33, B65) only elicited a weakly positive crossmatch result on neat serum (105 median channel shift above the negative control serum; positive T cell cutoff is >70 median channels), which was rendered negative (44 median channel shift) at a 1:2 serum dilution (Figure 10C). Similar results were seen with the B cell crossmatches (not shown), consistent with the presence of low-level class I HLA DSA. In contrast, AXE studies using the same surrogate donor cells showed a clear pattern of adsorption in a donor specific manner (HLA-A2, A33 and B65) in eluates performed with all serum concentrations from neat to a 1:4 dilution (Figure 10B). Therefore, while both FCXM and AXE confirmed the presence of low-level HLA antibody in the serum, the AXE protocol was able to verify the predicted antibody specificities correlating to the immunizing event, and appeared to exhibit greater sensitivity for detection of HLA antibodies.

## Discussion

The inability to distinguish clinically relevant anti-HLA antibodies from spurious artifacts on the SAB test is a prevalent and serious challenge. Currently, HLA laboratories investigate suspicious reactivities *via* redundant testing on alternative solid phase platforms (i.e. OneLambda vs Immucor), re-testing on phenotype beads which are coated with native HLA proteins rather than recombinant molecules, and performing surrogate flow crossmatches as a functional read-out. This multi-testing approach is costly, lengthens turn-around-time, and frequently does not provide adequate confidence in antibody calling to meet the clinical needs. For transplant programs, cautious listing of spurious HLA antibodies as unacceptable antigens can unfairly deny life-saving transplant opportunities for waitlisted candidates. When performed post-transplant, imprecise definition of donor-specific antibodies can confound the diagnosis of antibody-mediated rejection and lead to erroneous treatment decisions.

In this study, we present a novel adsorption with crossmatch cells and elution (AXE) procedure as an investigative tool to distinguish clinically meaningful HLA antibodies that target native HLA molecules from those binding to denatured antigens as well as other non-specific/background reactivities that can occur in the SAB assay. We based our assay design on the adsorption/elution technique which is routinely used in transfusion medicine and could be rapidly adapted for clinical grade testing in the HLA laboratory. In 2007, El-Awar first described the isolation of HLA antibodies using adsorption-elution with recombinant single HLA antigen cell lines and testing the eluate with the SAB assay (El-Awar et al., 2007; El-Awar et al., 2017). This method accurately defined HLA antibody specificity and helped characterize HLA epitopes. Our results confirm these findings and extend them by showing that adsorption/elution can also be used to distinguish antibodies binding to native HLA antigens from those binding cryptic epitopes on denatured molecules. When the adsorption/elution technique is coupled with carefully selected target donor cells, the AXE protocol can preserve the sensitivity of the SAB assay without compromising its specificity.

In the first part of the AXE assay evaluation we demonstrated the exquisite sensitivity of AXE in detecting antibodies not only against native HLA antigens expressed on donor cells, but also those binding to third party HLA alleles which share the same donor-specific epitope(s). Moreover, the results show that AXE can adsorb/elute antibodies with similar efficiency along a wide continuum of MFIs, and is more sensitive than the FCXM. These findings support the feasibility of using AXE to investigate suspicious low-level reactivities seen with the SAB assay, which could not be achieved using less sensitive phenotype bead panels or surrogate crossmatches. The AXE test thus fills an important gap as the standard SAB test is unable to discriminate low level reactivities which are at risk of immune memory/injury from spurious signals in the background. When AXE is used together with eplet analysis, we showed that the assay can isolate clinically relevant antibody reactivities in complex sera that correspond to mismatched HLAs from the immunizing events. As the indications for evaluating eplet incompatibility expands in transplantation, the AXE assay could further serve as an important tool to investigate both non-antibody-verified and novel epitopes as shown in the example highlighted in Figure 8.

One major benefit of the AXE assay is its ability to discriminate false positive reactivities on the SAB that clearly show no evidence of binding to native HLA. Multiple studies have consistently described the high prevalence of unexplained antibody reactivities on the SAB test in non-sensitized individuals. In an early study, 63% of non-sensitized male blood donors had detectable HLA antibodies on the SAB platform (Morales-Buenrostro et al., 2008). More recently, two studies reported close to 80% of wait-listed kidney patients without a history of sensitization tested positive on the solid phase assay (Gombos et al., 2013; Lan et al., 2020), with many reactivities being directed against common alleles in the population. In addition, using a modified SAB preparation (iBeads) which is mostly devoid of denatured class I HLA, it was shown that preformed donor-specific antibodies against denatured HLAs are clinically irrelevant (Otten et al., 2013). Visentin et al. further characterized the difficulty of distinguishing such spurious reactivities from genuine HLA antibodies in highly sensitized individuals (Visentin et al., 2015). In our study we demonstrated the ability of AXE to prevent indiscriminate listing of clinically insignificant antibodies as unacceptable antigens. Given the improved sensitivity and specificity of AXE compared to the FCXM, this technique could be further evaluated in future studies to determine its utility as a replacement assay for pretransplant crossmatches which currently have a false positive rate as high as 10–20% (Bachelet et al., 2016; Johnson et al., 2016; Sullivan et al., 2018). When paired with the rapid SAB assay (Liwski et al., 2017), the AXE protocol can be performed in less than 2 hours, which would represent a significant reduction in turn around time compared to the FCXM assay.

The use of appropriate controls is key to ensuring the validity of the AXE procedure results. When studying sera suspected to contain spurious/non-specific reactivity, it is important that a positive control serum is included to demonstrate that corresponding antibody specificities are efficiently adsorbed and eluted using the donor cells. In addition, testing of the supernatant that remains after the final wash following adsorption (last wash control) is critical to ensure no unbound antibodies are left after the cell washes, prior to antibody elution. This is especially important when adsorbing sera containing high level HLA antibodies. In our experience and based on the data presented in this study, six washes are sufficient to eliminate virtually all residual antibodies. However, additional washing steps could be implemented during the initial protocol evaluation by other laboratories to ensure efficient washing.

As is the case with the standard SAB assay where a universal positive cutoff has not been established, we rely on bead reactivity, pattern analysis (including epitope and cross-reactive group analysis) and the comparison of bead reactivity between the original serum, the eluate, and the last wash when interpreting AXE test results. When performed correctly, the last wash control should be completely negative and any reactivity in the eluate that is also seen in the original serum can be considered positive.

The efficiency of the AXE protocol is likely dependent on many factors including the initial antibody level, antibody affinity for the target antigen, the level of antigen expression on the donor cells, number of donor cells expressing the antigen, and assay conditions such as the initial serum volume and elution volume. The data presented in Figure 3 suggest that class II HLA antigens are adsorbed and eluted less efficiently compared to class I HLA. This is likely related to lower number of class II HLA expressing cells present in blood. Interestingly, our preliminary studies using donor lymphocytes isolated from spleens show improved efficiency for adsorbing/eluting class II antibodies, likely due to the increased number of B cells in the splenic lymphocyte preparations. Since multiple factors can affect the level of antibody absorption in the AXE protocol, the purpose of estimating the efficiency of absorption/elution in the current study was not to quantitate the absolute recovery of the antibody present in the original serum. Rather, we defined the efficiency of the AXE protocol as the percentage of the MFI obtained in the eluate as compared with the original serum to provide an estimate of assay sensitivity given the current protocol conditions.

The efficiency of adsorption/elution estimates are virtually identical when calculated using either raw or baseline MFIs for specificities above 300 MFI. Below this MFI level, baseline formula tends to underestimate the efficiency of adsorption/elution. Thus, in order to remain conservative in our estimations and because, in our experience, most HLA laboratories using LABScreen SAB kits use a baseline formula to account for background reactivity with the negative control bead and negative control serum, we elected to use baseline MFI values for analysis in this study. Additional testing using a large cohort of patients will likely be required to determine the choice of MFI units and if a reliable positive cutoff can be established to interpret results.

A potential limitation of the AXE protocol may be the availability of surrogate donor cells to perform the assay. Typing of staff members, flagging donor samples with HLA antigens of interest and cryopreservation of unused donor cells can be very useful in this regard. However, given that several alternative antigens can be used to adsorb/elute antibody patterns with the AXE protocol and the fact that having multiple mismatched antigens in a single donor is useful in order to fully interrogate reactivity patterns present in sera, donor availability for the AXE procedure is much less of an issue than the identification of a surrogate donor for FCXM, where a single, specific mismatch is required to assess the antibody reactivity in question. For this reason, the use of FCXM is not practical in assessing antibody patterns in highly sensitized patients as it is rarely possible to identify suitable surrogate donors expressing isolated HLA mismatches of interest. The AXE method described in the current study uses a tube technique, which requires a large amount of donor cells and allows only up to 3 samples (plus a positive control) to be comfortably processed at the same time. To overcome this limitation, we are currently validating a tray method for the AXE assay. Our preliminary experience with the tray-based protocol suggests an excellent efficiency of adsorption/elution and the ability to process 10–15 samples simultaneously. Development of the tray method will be very important in evaluating the possibility of using the AXE protocol as a replacement for the FCXM assay, where higher throughput will be required.

In conclusion, we have developed and optimized an adsorption/elution protocol which improves upon the specificity of the standard SAB test for antibody detection. Implementation of this procedure has the potential to improve candidates’ access to transplantation and add precision to post-transplant donor specific antibody monitoring.

## Data Availability

The original contributions presented in the study are included in the article/supplementary material, further inquiries can be directed to the corresponding author.
